# Very-Low-Dose Levodopa Therapy for Pediatric Neurological Disorders: A Preliminary Questionnaire in Japan

**DOI:** 10.3389/fped.2021.569594

**Published:** 2021-03-04

**Authors:** Kyoko Hoshino, Masaharu Hayashi, Asayo Ishizaki, Kazue Kimura, Masaya Kubota, Atsuo Nezu, Akihiro Yasuhara

**Affiliations:** ^1^Segawa Memorial Neurological Clinic for Children, Tokyo, Japan; ^2^The Very-Low-Dose Levodopa Therapy Research Group http://www.segawa-research.com/ldopa-therapy/index.html, Tokyo, Japan; ^3^Department of Pediatrics, Minami Wakayama Medical Center, Wakayama, Japan; ^4^School of Nursing, College of Nursing and Nutrition, Shukutoku University, Chiba, Japan; ^5^Oji Clinic, Division of Medicine, The Japanese Association on Intellectual and Developmental Disorders, Tokyo, Japan; ^6^Division of Neurology, National Center for Child Health and Development, Tokyo, Japan; ^7^Yokohama Medical and Welfare Centre, Kanagawa, Japan; ^8^Yasuhara Children's Clinic and YCC Education Center, Osaka, Japan

**Keywords:** very low dose levodopa therapy, autism spectrum disorder, tics, tourette syndrome, attention-deficit/hyperactivity disorder, dopamine receptor supersensitivity, Rett syndrome

## Abstract

**Introduction:** Post-synaptic dopamine receptor supersensitivity (DARSS) has been extensively researched by Dr. Masaya Segawa, who has investigated the efficacy of very-low-dose levodopa therapy (VLDT; 0.5–1 mg/kg/day). Considerable Japanese research supports the possibility that VLDT could be used to treat pediatric neurological disorders. We conducted an on-line survey in 2014 to collect real-world data on the use of VLDT to treat DARSS.

**Methods:** A two-step survey, including a screening test and questionnaire, was posted on a private internet site that could be accessed via the VLDT Research Group home page, and 1,165 pediatric neurologists across Japan were invited to complete it.

**Results:** A total of 25 respondents reported prescribing VLDT; 19 used VLDT to treat autism spectrum disorder, 14 for tics, 12 for speech delay, 9 for Rett syndrome, 7 for attention-deficit/hyperactivity disorder, intellectual disability, and 6 for sleep problems. Twelve respondents reported prescribing a dose of 0.5 mg/kg. Twenty-two reported that VLDT was effective for treating behavioral problems, and twenty reported a good efficacy for treating motor symptoms. Adverse events had a low incidence. Notably, respondents chose VLDT for its possible action in DARSS and for its safety. VLDT was commonly used for behavioral problems in patients younger than 5 years, and for motor symptoms in aged 5–9 years.

**Conclusion:** VLDT could safely treat behavioral and motor symptoms in pediatric neurological disorders. In contrast, dopamine antagonists are associated with potent efficacy, but with adverse effects such as sleepiness and obesity. Further surveys should be conducted with a broader participants.

## Introduction

Post-synaptic dopamine receptor supersensitivity (DARSS) is an epiphenomenon of neuronal denervation that has been associated with multiple neurodevelopmental disorders and other pediatric neurological conditions (1–6). DARSS has been extensively researched by Dr. Masaya Segawa, who has examined the possibility of treating it with very-low-dose levodopa therapy (VLDT) (0.5–1 mg/kg/day). His work has substantiated the use of VLDT in the treatment of pediatric DARSS-associated disorders (7, 8). VLDT differs from low-dose levodopa treatment for Segawa disease (SD), in that the levodopa dose for Segawa disease treatment is 4–10 mg/kg/day, whereas that of VLDT is extremely small (9).

In 1983, Dr. Shigeki Tanaka, first described the use of VLDT to treat DARSS in an 11-year-old girl with tuberous sclerosis and a nodule in the left thalamostriatal sulcus (10). The patient exhibited infantile spasms, autistic features, and rotatory seizures on the right side. Her seizures worsened with a 4-mg/kg dose of levodopa, a dopamine antagonist improved the seizures but suppressed movement during sleep, and a VLDT of 0.5 mg/kg improved seizures while also normalizing movement during sleep. Subsequent sleep studies in patients with pediatric sleep movement disorders have revealed the involvement of hypoactive dopamine transmission, which is suggestive of a pathophysiological mechanism of dopamine dysfunction in the nigrostriatal pathway via the corticospinal tract (7, 8, 11, 12). Dopamine antagonists improved rotatory seizures but suppressed daytime movement and caused abnormal movement during sleep. Only a VLDT of 0.5 mg/kg improved rotatory seizures and resulted in almost normal movement during sleep, which supports the hypothesis that VLDT stabilizes DARSS and normalizes dopamine transmission.

VLDT has shown promise as an effective treatment for autism spectrum disorder (ASD) (13, 14), Tourette's syndrome (15), Rett syndrome (RTT) (12, 16), and laryngeal dystonia in patients with xeroderma pigmentosum (17). The greatest benefit of VLDT is thought to be its safety, and the second point is the understandable explanations of its mechanism of action in treating DARSS. Most of Dr. Segawa's colleagues went on to use and teach other colleagues about VLDT all over Japan. Thus, a small number Japanese child neurologists also hope that VLDT might have a good efficacy in treating intractable diseases. However, a double-blinded crossover study with 20 patients with ASD failed to find a significant efficacy of VLDT in Japan (13). That said, four cases (20%) showed an improvement in humor, mood, social activity, eye contact, and language.

In this study, we investigated why these Japanese pediatric neurologists used VLDT, because there is little evidence that this well-known medication has any efficacy in treating intractable diseases. While the existing results indicate that this treatment is promising, there is scarce information on VLDT's clinical efficacy, adverse effects, optimal dosing, or indications. We thus devised a two-step questionnaire to collect real-world data on the use of VLDT. VLDT has been used only in Japan, because levodopa powder is not available in other countries. However, levodopa tablets crushed into powder could be used, which means that trials to assess the efficacy of VLDT in patients with developmental disorders could be performed worldwide.

## Methods

This survey was initiated in 2014, and comprised a screening questionnaire and a main questionnaire that were developed by members of the VLDT Research Group (18). The screening questionnaire was posted on a private internet site that could be accessed via the VLDT Research Group home page, and pediatric neurologists across Japan were invited to complete it. The invitations were sent through professional mailing lists, including the editorial board of the Society of Child Neurology in Japan (*n* = 200), a private mailing list of pediatric neurologists in Japan (*n* = 820), the Child Sleep Net of Japan (*n* = 70), and the Pediatric Net of Wakayama (*n* = 75). Participants responded to the survey online and selected their responses from predefined options. Multiple answers were permitted for Q2.1 and Q2.2, whereas only one answer was allowed for each of the other questions. If no response was appropriate, the respondent was permitted to skip that question. Respondents could add comments to any survey item. The screening survey included basic questions on VLDT use (Supplementary Table 1). The responses for Q1.2, 1.3, 1.5, 1.6, 1.7, and 1.8 were classified into 4 groups according to the number of patients treated with VLDT, as follows: (A) fewer than 5 patients, (B) 5–9 patients, (C) 10–19 patients, (D) 20 patients or more.

The main survey was sent to respondents who had reported using VLDT in the screening survey. The main survey requested detailed information on the use of VLDT, as shown in Supplementary Table 2. VLDT is not currently an approved treatment in Japan, so the use of VLDT was reviewed and approved by the ethical committee of the Neurological Clinic for Children (No. NCC-01) prior to the study and all research was in compliance with the Neurological Clinic for Children guidelines. Due to the nature of the surveys, it was not possible to obtain consent from patients. However, personal information was neither revealed nor used in this paper. The pediatric neurologists who participated in the survey consented to the use of their responses in our research. The first author takes complete responsibility for the integrity of the data and accuracy of the data analysis.

## Results

A total of 51 pediatric neurologists responded to the screening questionnaire. A link to the main questionnaire was sent to the 25 (49%) neurologists who reported using VLDT; of these, 18 responded.

### Screening Questionnaire

#### Target Disorders and Symptoms

**Figure 1:**
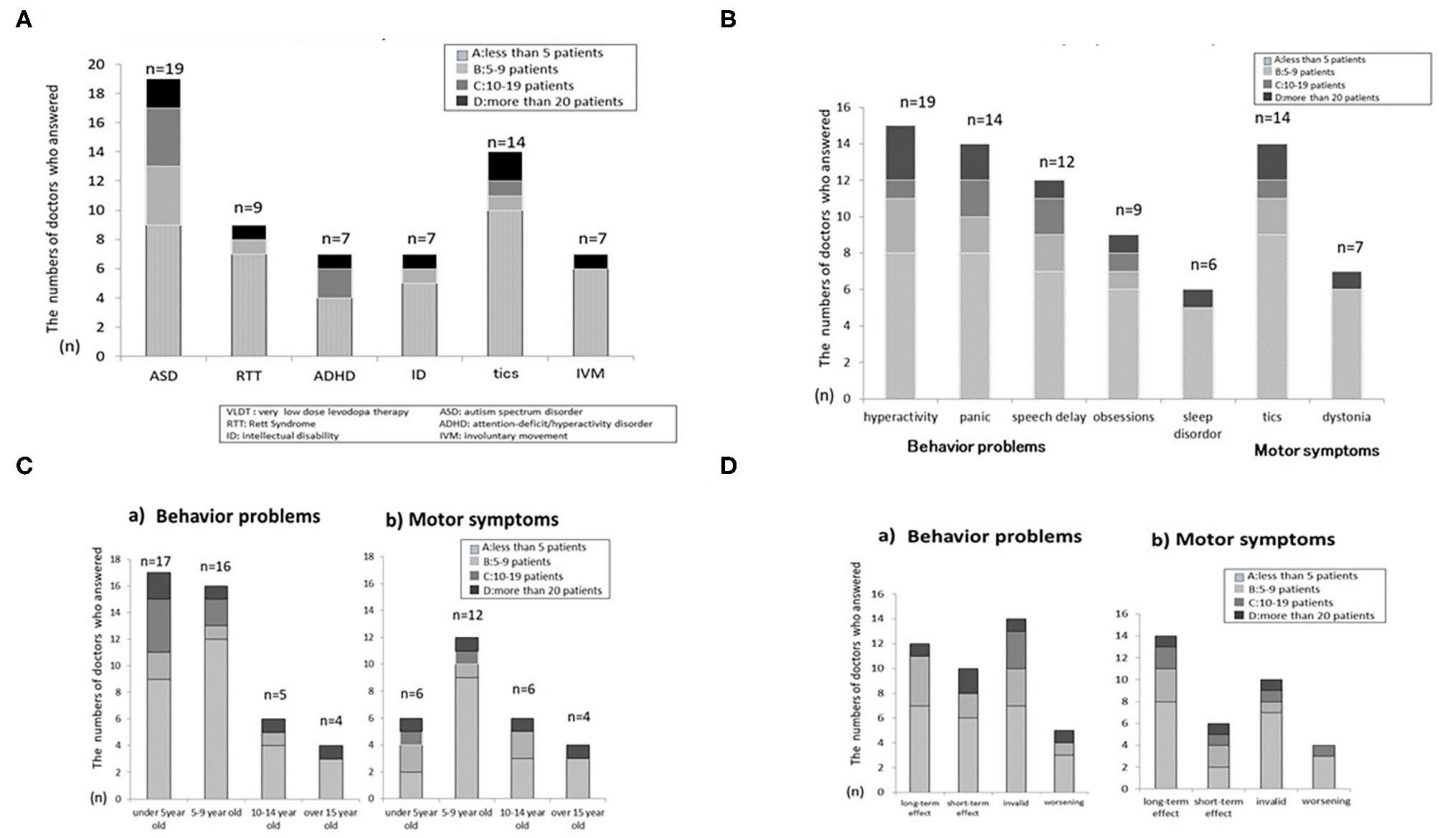
**(A)** Q1.2 What diseases do you treat with VLDT? **(B)** Q1.3 For what symptoms do you use VLDT? **(C)** Q1.5 How old are the patients for whom you prescribe VLDT? **(D)** Q1.6 Please describe the effectiveness of VLDT.

Of the 51 respondents of the screening questionnaire, 25 reported prior use of VLDT, 20 (39%) had not used VLDT, and 6 (12%) had no knowledge of VLDT. Among the 25 respondents who had used VLDT, 19 (76%) reported use for ASD, 14 for tics, 9 for RTT, 7 for attention-deficit/hyperactivity disorder (ADHD), 7 for intellectual disability (ID), and 7 for involuntary movement (Figure 1A). The use of VLDT was also reported for restless leg syndrome and sleep problems (nocturnal awakening) by two pediatric neurologists, and stuttering and ASD-associated social-skill problems by one neurologist. A total of 19 respondents used VLDT for irritability, 12 for speech delay, 9 for insistence of sameness, 6 for sleep problems, and 7 for dystonia, including an initial treating dose for Segawa Disease and other dystonia syndrome as DYT1 (Figure 1B). Respondents also used VLDT to improve social activity, eye contact, verbal function (comprehension, expression), mood, motivation, activity, and/or fine-motor skills.

#### Dosage, Patient Age, and VLDT Efficacy

Of the 25 respondents who had experience with VLDT, 12 reported using a VLDT dose of 0.5 mg/kg. Of the remaining 14 respondents, 12 used 0.5–0.9 mg/kg, 8 used 0.3 mg/kg, 4 used 0.1 mg/kg, and 1 used 1 mg/kg. Notably, some physicians reported using multiple dosages for different purposes.

Target symptoms were classified as either behavioral or motor. The age distribution of patients in relation to these two categories is shown in Figure 1C. In both symptom categories, most physicians used VLDT for children younger than 10 years; VLDT was most widely used for behavioral problems in patients younger than 5 years and for motor symptoms in patients 5–9 years of age (Figure 1C). Findings regarding VLDT efficacy are shown in Figure 1D. Some neurologists had multiple answers for this questionnaire item due to the variety of outcomes they observed in their practice. For the treatment of behavioral symptoms, 12 neurologists reported long-term efficacy and 10 reported short-term efficacy, mostly selecting “ < 5 patients” or “5–9 patients” in their responses, while 14 reported that VLDT was not effective. For motor symptoms, 14 neurologists reported long-term efficacy, 6 reported short-term efficacy, and 10 reported that VLDT was not effective. A few instances of worsening behavioral and motor symptoms were reported (Figure 1D).

#### Concomitant Drugs

Use of concomitant drugs is shown in Supplementary Figure 1. Methylphenidate, atomoxetine, risperidone, haloperidol, aripiprazole, and selective serotonin-reuptake inhibitors were prescribed as concomitant drugs for behavioral symptoms; risperidone, haloperidol, and aripiprazole were prescribed for motor symptoms. Additional drugs included anticonvulsants (carbamazepine, valproate acid, and gabapentin), melatonin, tandospirone, and Chinese herbal medicines for behavioral problems; pimozide, clonidine, and clonazepam for tics; and trihexyphenidyl hydrochloride for involuntary movement.

#### Adverse Events

**Figure 2:**
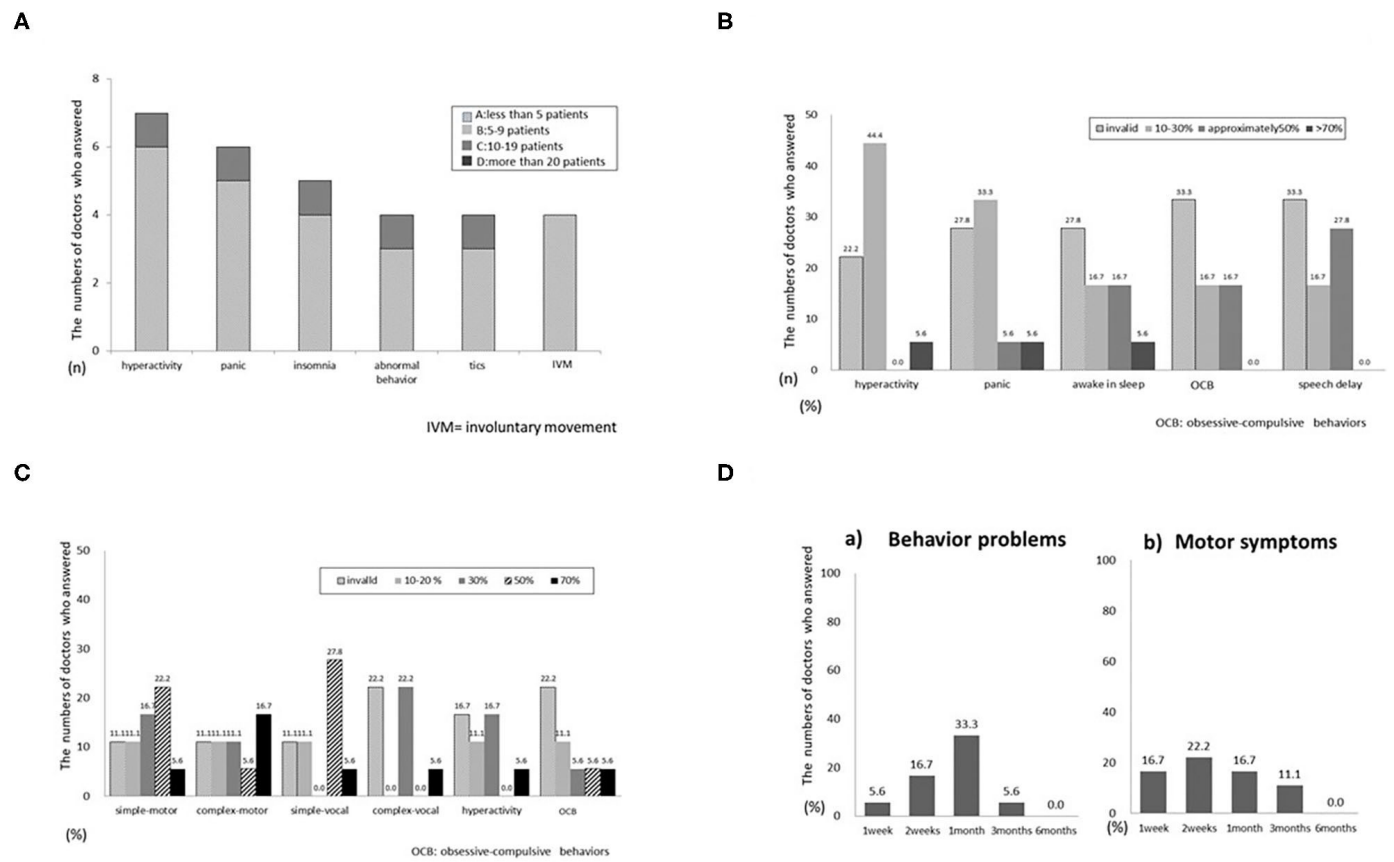
**(A)** Q1.8 What adverse events have you observed? **(B)** Q2.3 Effectiveness for specific symptoms of autism spectrum disorder (ASD). **(C)** Q2.7 Effectiveness for specific symptoms of tics. **(D)** Q2.9 When was the effectiveness of VLDT observed?.

Adverse events were reported by 4 neurologists and included hyperactivity, pain, insomnia, abnormal behavior, tics, and involuntary movement (Figure 2A). Most events were of a low incidence (fewer than 5 patients). Adverse events included polyuria, dizziness, headache, constipation, and irritability.

### Main Questionnaire

#### Reasons for VLDT Use and Indications

Eighteen pediatric neurologists who answered the first online questionnaire subsequently responded to the second questionnaire, which requested additional details on VLDT use (Supplementary Table 2). Responders could answer questions multiple times to represent the range of effects of VLDT seen in their clinical practice. When asked why they prescribed VLDT, responders could choose one or more answers to reflect their clinical practice. Eleven of the eighteen respondents selected “for possible DARSS,” ten selected “safety,” and three selected “because of the adverse effects of dopamine antagonists.” One-third of respondents referred to Segawa's early research.

#### VLDT Efficacy by Disease

The main questionnaire also requested information on VLDT efficacy in relation to disease symptoms. For ASD (Figure 2B), efficacy varied from mildly effective to ineffective for all symptoms. Most respondents reported that VLDT was ineffective for all symptoms of RTT (Supplementary Figure 2A). For ADHD (Supplementary Figure 2B), 4 neurologists reported efficacy in 10–30% of patients with hyperactivity. Three reported efficacy in 10–30% of patients with irritability. Only a few respondents reported that VLDT was more than 50% effective for nocturnal awakening or was effective for symptoms of intellectual disability (Supplementary Figure 2C). Most respondents reported that VLDT was 30–50% effective for all types of tics (Figure 2C), but not for hyperactivity or insistence of sameness. VLDT was more effective for simple tics than for complex tics and was ineffective for all types of involuntary movement (Supplementary Figure 2D). Only 1 respondent reported an efficacy > 70% for tremor.

#### Interval to Treatment Effects and Adverse Events

The interval from the start of VLTD to initial treatment effects ranged from 2 weeks to 1 month for motor and behavioral symptoms (Figure 2D). Five respondents reported an interval of < 1 week from the start of VLTD to the onset of adverse events. Four reported adverse events within 2 weeks, and one reported adverse events within 1 month. When adverse events occurred, 3 respondents stopped VLDT immediately, 2 suspended treatment and later resumed at the same dose, and 2 suspended treatment and resumed at a lower dose. None of the respondents continued uninterrupted VLDT after patients developed adverse effects. The final question addressed concomitant medicines, particularly those used in conjunction with VLDT; 3 respondents reported other drug treatment concomitant with VLDT.

## Discussion

This is the first screening survey to assess VLDT use in Japan. Our aim was to clarify how VLDT is used in clinical practice by Japanese pediatric neurologists. Among the respondents, 49% were aware of VLDT and used it in their clinical practice. These physicians reported that VLDT was effective in 30–50% patients treated for nocturnal awakening and tics but was minimally effective or ineffective for the treatment of other disorders.

In the screening questionnaire, VLDT use was reported for diseases and symptoms considered to result from DARSS, including ASD (5, 13, 14), tics (15), and RTT (12), and for symptoms of hyperactivity, irritability, and speech delay (5, 14). The most common VLDT doses was 0.5 mg/kg, which is the dose first reported by Segawa, and 0.5–0.9 mg/kg. Further investigation into the most effective dosages is needed. VLDT was most widely used for behavioral problems in patients younger than 5 years and for motor symptoms in patients aged 5–9 years. This finding may be due to changes in dopamine levels and receptor activity seen throughout childhood. Indeed, the production of dopamine changes remarkably from early childhood to the age of 20 years, because tyrosine hydroxylase activity declines with age (19). Dopamine activity and dopamine receptor sensitivity may also change with age, and DARSS may therefore manifest throughout childrens' development (19–21). This physiological factor may explain the difference in the efficacy of VLDT as patients get older.

Responses to the main questionnaire suggest that VLDT is mildly effective for ASD and ADHD, but not for RTT or intellectual disability, possibly because clinical impairment was more severe in the latter two categories. Few doctors reported treatment-related improvements in attention, motivation, or humor in patients with ASD. VLDT was effective for simple and complex tics, but not for any other type of involuntary movement. This suggests that tics in young children may result from DARSS, although other involuntary movements may be caused by other pathologies. The effects of VLDT were apparent within 1 month after the start of therapy.

Previous reports concerning dopaminergic function in neurodevelopment disorders could explain the effect of VLDT on psychiatric features, tics, and restless legs syndrome. In one review, Hallett (22) reported that Tourette's syndrome is associated with a developmental hypofunction of dopamine neurons that leads to dopamine receptor hypersensitivity. Avery low dose of levodopa has also been reported as an initial treatment in infants with tyrosine hydroxylase deficiency (23). Restless legs syndrome is well-known to initially respond to levodopa and iron; while a regular dose of levodopa has been found to induce dopaminergic augmentation (24), VLDT did not result in such an augmentation in children (25).

The British Association for Psychopharmacology has reviewed the treatment of symptoms of ASD (26), and noted the potential of treatments that are similar to VLDT.

Namely, antipsychotics (dopamine antagonists) and methylphenidate can alter dopaminergic receptors; however, the former suppresses and the latter activates dopaminergic activity. While this may seem contradictory, hypo-dopaminergic activity can induce DARSS in the early stages of neuronal denervation (1–4). We believe that DARSS should be a focus in investigations into the pathophysiology of neurodevelopmental disorders, and that VLDT could normalize dopamine transmission.

Most existing studies have reported the statistical results of each medication for neurodevelopmental diseases such as ASD, ADHD, and tics, but have rarely examined activity at the synaptic site of dopaminergic neurons following VLDT treatment. Dr. Segawa has proposed that the action of VLDT on DARSS can result in improvements in symptoms related to dopamine transmission.

This study has a number of limitations. First, only 51 pediatric neurologists participated in the internet-based questionnaire, and these were almost certainly skewed toward physicians who were familiar with the internet and interested in online surveys. Second, the initial ratio of respondents (51/1,165) was small, which indicates that there may have been a bias toward physicians who were interested in VLDT; however, the response rate to the main questionnaire was remarkably high (18/25).

To overcome these limitations, further surveys should be conducted with more participants and with a broader cross-section of pediatric neurologists. Despite these limitations, the present study provides a useful starting point for further discussion of the use of VLDT in the treatment of neurodevelopment disorders in pediatric patients. A randomized controlled trial will be required for more definitive results.

In summary, VLDT is thought to be mildly effective for mild cases of DARSS, and rarely causes adverse effects. In contrast, dopamine antagonists are associated with a potent efficacy, but have severe adverse effects, including sleepiness, and weight gain. In addition, most dopamine antagonists decrease higher cortical functions. The comparative safety of VLDT is therefore attractive. Additionally, aripiprazole, which stabilizes dopamine receptors, has recently been reported to effectively treat irritability in ASD (26, 27). In the future, treatment might be selected according to disease severity. The efficacy of combined VLDT and aripiprazole could be investigated in future studies.

## Data Availability Statement

The raw data supporting the conclusions of this article will be made available by the authors, without undue reservation.

## Ethics Statement

The studies involving human participants were reviewed and approved by the ethical committee of the Neurological Clinic for Children (No. NCC-01). Written informed consent to participate in this study was provided by the participants' legal guardian/next of kin.

## Author Contributions

KH research project conception and execution, integrity of the data and accuracy of the data analysis, manuscript preparation, writing of the first draft, review, and critique. MH manuscript preparation, review, critique, and restructuring of the manuscript. AI, KK, MK, AN, and AY manuscript preparation, review, and critique. All authors contributed to the article and approved the submitted version.

## Conflict of Interest

The authors declare that the research was conducted in the absence of any commercial or financial relationships that could be construed as a potential conflict of interest.

## Supplementary Material

Supplementary Figure 1 Q1.7 What concomitant drugs have you prescribed?

Supplementary Figure 2 **(A)** Q2.4 Effectiveness for specific symptoms of Rett syndrome (RTT). **(B)** Q2.5 Effectiveness for specific symptoms of attention deficit hyperkinetic disorder (ADHD). **(C)** Q2.6 Effectiveness for specific symptoms of intellectual disability (ID). **(D)** Q2.8 Effectiveness for specific symptoms of involuntary movement (IVM).

Supplementary Table 1 1st Questionnaires.

Supplementary Table 2 2nd Questionnaires.
