# Supplementary figures and images for: Very-Low-Dose Levodopa Therapy for Pediatric Neurological Disorders: A Preliminary Questionnaire in Japan
Source: Front Pediatr. 2021 Mar 4;9:569594. doi: 10.3389/fped.2021.569594 (PMC7970027; doi:10.3389/fped.2021.569594)

## Slide 1
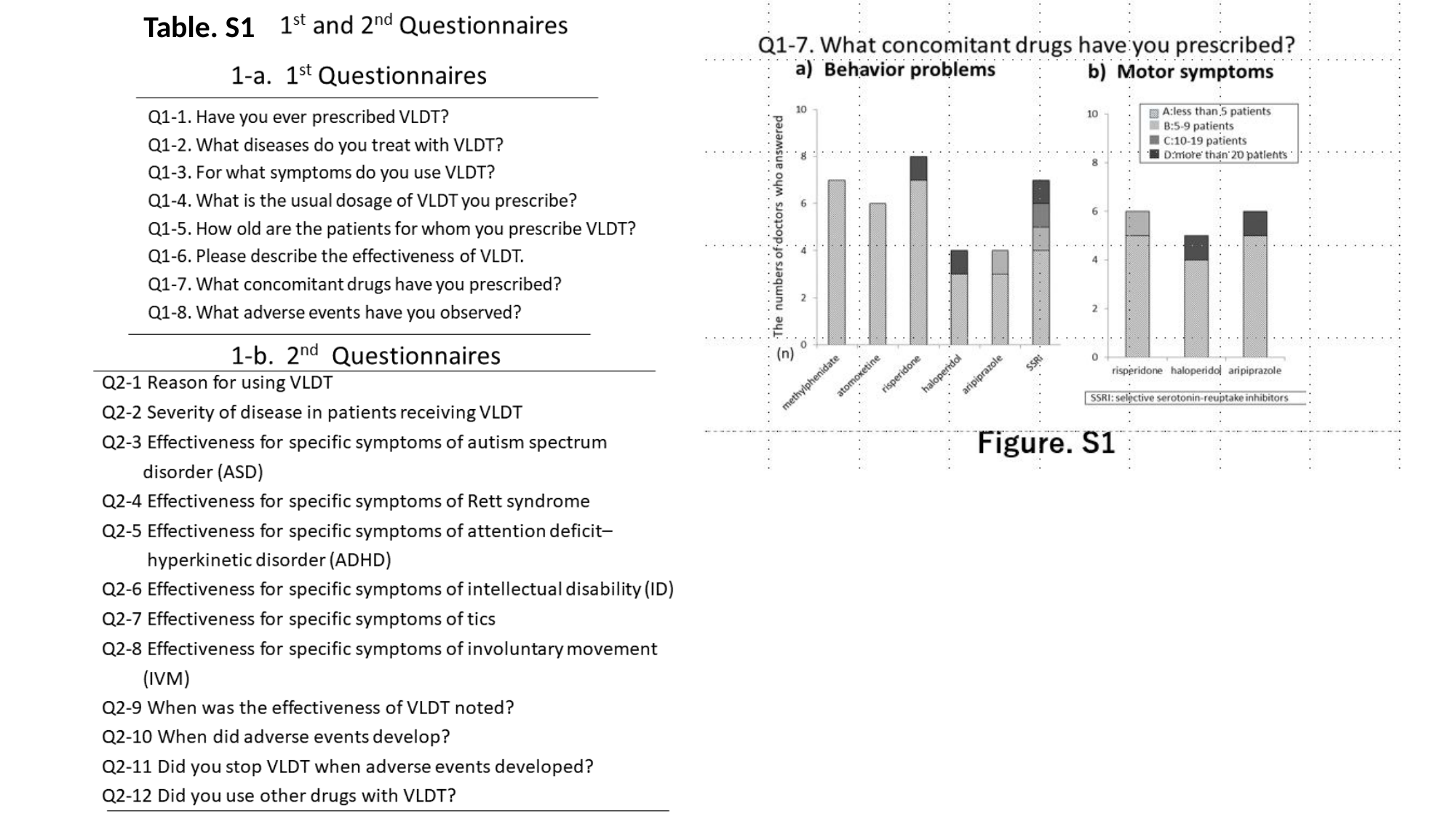

Table. S1
#

## Slide 2
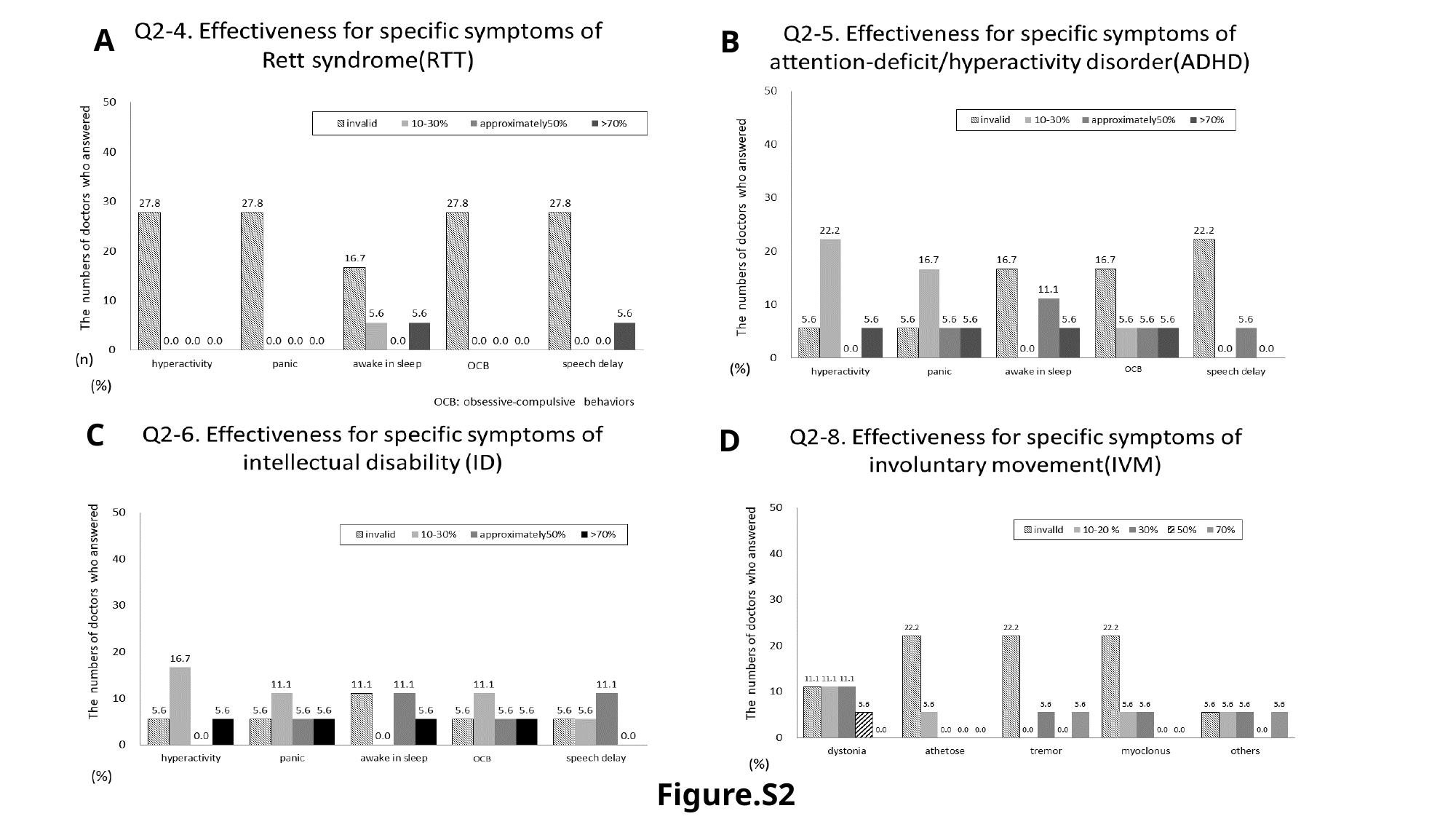

A
B
C
D
Figure.S2

Supplement: Supplementary Figure 1 — Q1.7 What concomitant drugs have you prescribed? [file Presentation_1.PPTX]
